# Biomimetic cellular vectors for enhancing drug delivery to the lungs

**DOI:** 10.1038/s41598-019-55909-x

**Published:** 2020-01-13

**Authors:** Michael Evangelopoulos, Iman K. Yazdi, Stefania Acciardo, Roberto Palomba, Federica Giordano, Anna Pasto, Manuela Sushnitha, Jonathan O. Martinez, Nupur Basu, Armando Torres, Sarah Hmaidan, Alessandro Parodi, Ennio Tasciotti

**Affiliations:** 1 Center for Biomimetic Medicine, Houston Methodist Research Institute, Houston, TX USA; 2 Department of Mechanical and Aerospace Engineering, Politecnico di Torino, Turin, Italy; 3 CEINGE Biotecnologie avanzate, Naples, Italy; 4 School of Medicine and Surgery, University of Milano-Bicocca, Monza, Italy; 5 Veneto Institute of Oncology–IRCCS, Padua, Italy; 6 Department of Bioengineering, Rice University, Houston, TX USA; 7 Institute of Molecular Medicine, Sechenov First Moscow State Medical University, Moscow, Russia; 8 Houston Methodist Orthopedic and Sports Medicine, Houston Methodist Hospital, Houston, TX USA

**Keywords:** Biomedical engineering, Drug delivery

## Abstract

Despite recent advances in drug delivery, the targeted treatment of unhealthy cells or tissues continues to remain a priority. In cancer (much like other pathologies), delivery vectors are designed to exploit physical and biological features of unhealthy tissues that are not always homogenous across the disease. In some cases, shifting the target from unhealthy tissues to the whole organ can represent an advantage. Specifically, the natural organ-specific retention of nanotherapeutics following intravenous administration as seen in the lung, liver, and spleen can be strategically exploited to enhance drug delivery. Herein, we outline the development of a cell-based delivery system using macrophages as a delivery vehicle. When loaded with a chemotherapeutic payload (i.e., doxorubicin), these cellular vectors (CELVEC) were shown to provide continued release within the lung. This study provides proof-of-concept evidence of an alternative class of biomimetic delivery vectors that capitalize on cell size to provide therapeutic advantages for pulmonary treatments.

## Introduction

Current targeting approaches rely primarily on physical and biological features of unhealthy tissue that differ from healthy tissue. However, biological heterogeneity goes beyond simplification into categories of healthy and unhealthy tissue or cells. For example, common characteristics of cancer disease (i.e., neo-angiogenesis^1^, enhanced permeability and retention^2^, inflammation^3,4^ and surface protein markers^5^) are not always homogeneous in neoplastic lesions^6^, thereby making the design of targeted delivery systems challenging. In addition, current efforts dedicated to manipulating the nanocarrier surface to match the biological complexity of the biological environment are often ineffective due to the mononuclear phagocyte system’s (MPS) ability to identify and sequester foreign materials^7,8^, leading to poor therapeutic efficacy. In parallel with the development of targeted platforms, other pharmacological interventions (i.e., adjuvant therapies) are intentionally administered to perfuse the entire organ affected by the tumor in an effort to combat the micro-infiltration of cancerous cells into the surrounding tissue^9^. Although these cells are not targeted with traditional nanocarrier surface modifications, they still represent a serious threat to the patient’s life. In this scenario, drug delivery systems can be designed to improve the efficacy of these therapeutic approaches while minimizing toxicity.

It has previously been shown that the organs that comprise the MPS disproportionally sequester injected delivery vehicles^10^, thereby showcasing the need for novel organ-specific interventions. It is commonly accepted that initial accumulation following the intravenous administration of a drug delivery system occurs within the pulmonary environment^11^. Pulmonary accumulation and clearance of a delivery vehicle have been shown to be primarily driven by the size of the carrier, with micron-sized systems displaying an increase in residence time compared to nano-sized systems^12^. To design effective micron-sized platforms that exploit natural circulation, it is critical to select materials that are both biocompatible and biodegradable^13,14^. Previous work performed by our group has demonstrated the benefit of using syngeneic source material in the design of biocompatible carriers that also bestow the delivery vehicle with higher affinity towards inflamed sites^15–17^. Recent literature has demonstrated the potential in using easy-to-isolate circulating cells as drug delivery carriers for the treatment of pulmonary cancer lesions^18^. In this effort, drug delivery systems were generated through passive loading of chemotherapy into macrophages, exploiting their natural tropism for neoplastic lesions and their efflux pumps to deliver the drug in the desired site.

**Figure 1:**
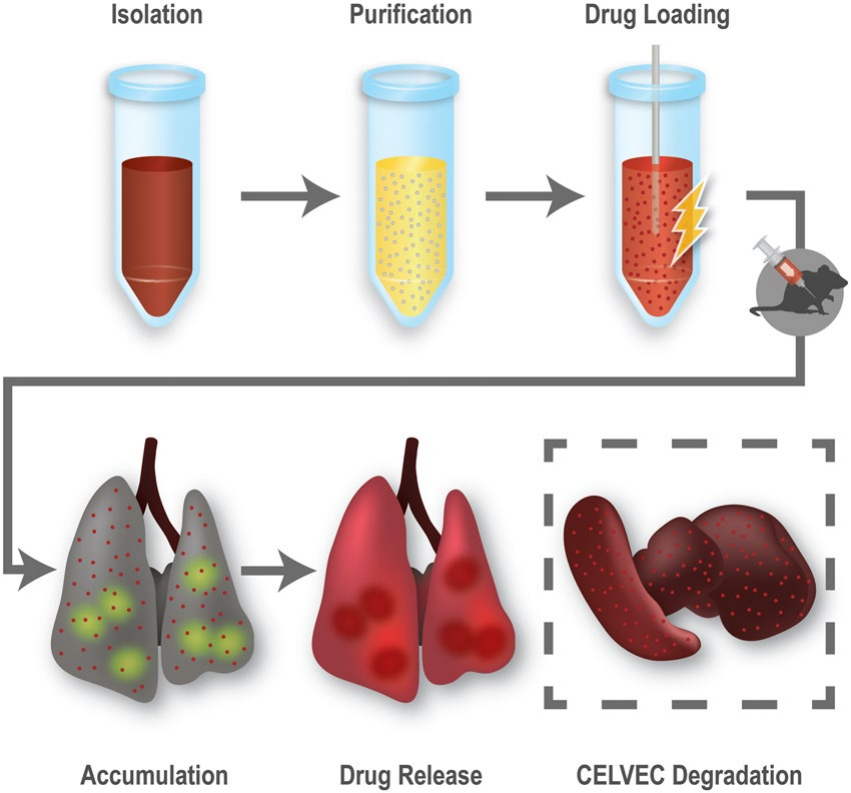
Schematic representation of CELVEC. CELVEC generated using leukocytes isolated from whole blood or grown using cell culture. Following purification, cells are loaded with a chemotherapeutic payload using electroporation. Once administered intravenously into a mouse model, CELVECs accumulate within the pulmonary environment or lungs. Payload is passively released within the first several hours of accumulation in a controlled fashion, following by CELVEC sequestration and degradation in the spleen and liver.

Herein, we defined the pulmonary residence properties of a macrophage cell-based delivery system and demonstrated favorable accumulation properties of a model chemotherapeutic payload, doxorubicin (DOX), into the lungs. However, compared to previous cell-based delivery platforms^18^, our system is composed of inactive cells loaded with a chemotherapeutic through electroporation, thereby enabling a more suitable approach for passive drug diffusion within the pulmonary environment. This work relies on the biomimetic exploitation of macrophages, previously pursued in our lab for drug delivery purposes^3,4,16^, thereby allowing us to shift the target from neoplastic tissues to whole organs. Through loading parameter optimization, we successfully generated cellular vectors (CELVEC, Fig. 1). This drug delivery strategy provides an advantageous delivery vector by allowing desirable spatiotemporal release of a chemotherapeutic payload (i.e., DOX) into the lungs with no observable acute side effects. More importantly, the system is based on a simple fabrication protocol that requires minimal training while providing substantial advantages regarding reproducibility, time, and costs that are known to hinder clinical translation of novel drug delivery technologies^19,20^.

## Materials and Methods

### Cell culture

J774A.1 murine macrophage cells were obtained from American Type Culture Collection (ATCC, Manassas, VA) and maintained using complete Dulbecco’s Modified Eagle Media (DMEM, HyClone) supplemented with 10% fetal bovine serum (Atlas Biologicals) and 1% Penicillin Streptomycin (Corning). The cells were maintained in a humidified Heracell 150i CO_2_ incubator (Thermo Scientific) under 37 °C and 5% CO_2_. All cells for CELVEC generation were maintained in a rolling bottle system to keep cells in continuous suspension.

### Fabrication of cellular vectors (CELVEC)

Electroporation Buffer containing doxorubicin (DOX, LC Laboratories) was created by slowly adding hydrochloric acid to obtain a final pH of 5.2. Next, DOX was added to the Electroporation Buffer (Teknova) to bring the working concentration to 20 mg/mL and briefly sonicated at room temperature to fully dissolve DOX. DOX-containing electroporation buffer was prepared fresh for all experiments. Characterization of DOX was performed using a Zetasizer Nano-ZS (Malvern Instruments Ltd.) instrument for measurement of size and polydispersity.

Prior to fabrication of CELVEC, cells were collected following a brief wash in phosphate buffered saline (PBS) and centrifuged at 125 × g for 10 min at room temperature. Following centrifugation, 2 × 10^4^ cells/μL were suspended in Electroporation Buffer supplemented with 20 mg/mL of DOX (unless otherwise stated) and transferred to electroporation cuvettes. Electroporation was performed using a 4D-Nucleofector System or Nucleofector 2b Device (Lonza) following the appropriate mouse macrophage program setting (<20 s of electroporation). Following electroporation, cells were placed in pre-warmed complete media and centrifuged at 125 × g for 10 min to remove unloaded DOX.

### Passive vs Electroporation

We compared the incorporation of passive and electroporation-mediated loading of DOX into J774 cells using flow cytometry. Briefly, 3 × 10^5^ J774 cells were electroporated as described above to create DOX@CELVEC. For passive loading, the same number of J774 cells were treated using the exact same conditions as electroporated but without being supplemented with electroporation and instead were incubated in microcentrifuge tubes. Samples for each condition were measured in triplicate using a BD LSRFortessa housed within HMRI’s Flow Cytometry Core. Samples were analyzed using the 561 nm excitation laser with PE-Texas Red filters applied. Control cells (i.e., no DOX) and CELVEC (i.e., electroporated but without DOX) were measured for background signal. Data from flow cytometry was exported and analyzed using FlowJo software.

### Drug loading and release estimation

Drug loading was performed as described above. Following electroporation, cells were provided 10 min of recovery time, then resuspended in DMEM cell culture media (without Phenol red) and allowed to incubate for 1 h at 37 °C. This was followed by centrifugation at 300 × g for 5 min with supernatant being removed and measured for fluorescence. Cells were then dispersed in RIPA buffer, briefly agitated, and analyzed for DOX fluorescence. This was compared to the total amount measured in the supernatant solution. All samples were measured for fluorescence using a Synergy H4 microplate reader set to excitation of 488 nm and emission of 588 nm.

Release from loaded cells was performed by subjecting cells to mild agitation with samples collected at predetermined time points. Briefly, cells were placed into microcentrifuge tubes and placed on a tube rotator providing consistent rotation. A small aliquot was collected and replaced with fresh media. Aliquot was compared to known DOX concentrations and added to previous time points. Fluorescence was measured as described above.

### Retention of macrophage surface markers after electroporation

We evaluated the retention of critical macrophage surface markers LFA-1 and Mac-1 after electroporation with and without DOX using Western Blot. Briefly, 5 × 10^6^ J774 control cells were prepared and electroporated in the absence or presence of DOX. Cells were washed three times in PBS and ran through an Amicon Ultra-0.5 Centrifugal filter (Millipore) to eliminate any residual free DOX. Cells were lysed with RIPA buffer supplemented with protease inhibitor cocktail. Protein concentration was determined using the micro bicinchoninic acid (micro-BCA) assay (Quantum Micro Protein, Euroclone). Equal protein amounts were loaded on 4–20% protein precast polyacrylamide gels (Biorad) in denaturing and reducing conditions. Proteins were then transferred onto nitrocellulose membranes (Biorad). Membranes were saturated with 5% non-fat milk in TBS-Tween buffer and hybridized overnight at 4 °C with the following anti-mouse antibodies: β-actin (1:1,000; Abcam), LFA-1 (1:500; Abcam), Mac-1 (1:500; Abcam). Finally, the chemiluminescence signal was detected with Western Lightning® Plus-ECL (Biorad) on a ChemiDoc™ XRS Imaging System.

### Electron microscopy

Scanning electron microscopy was performed by briefly washing the cells twice in PBS and fixed using 2.5% glutaraldehyde for 30 min. Cells were washed then dehydrated using increasing concentrations of ethanol (30, 50, 70, 90, 95, 100) for 10 min each. Samples were next exposed to 50% alcohol-hexamethyldisilazane (HDMS) for 10 min, following by 10 min incubation with HDMS alone. Following overnight drying, samples were mounted, and sputter coated with gold, followed by image acquisition using an FEI Quanta 400 FEG ESEM equipped with an ETD (SE) detector (FEI). Samples for high-resolution transmission electron microscopy were prepared similarly with image acquisition performed using a FEI Tecnai electron microscope.

### Neutral red uptake

Cellular viability was performed by seeding cells into a 96-well plate at a concentration of 4,000 cells/well and allowed to incubate overnight at 37 °C. Neutral red uptake analysis was performed following previously established protocols^21^. Briefly, cells were incubated for 2–4 h with DMEM cell culture media (without phenol red and serum) containing 50 mg/mL of neutral red dye. Following incubation, staining solution was discarded and cells were fixed for 10 min using 4% paraformaldehyde. Next, solubilization solution was added to the plate and subjected to brief agitation on a plate rocker for 10 min at room temperature. Absorbance at 540 nm was then read using a BioTek Microplate reader (BioTek Instruments).

### Apoptotic evaluation

Evaluation of apoptosis was performed using a FITC Annexin V Apoptosis Detection Kit I (BD Biosciences) in combination with flow cytometry. Briefly, CELVEC generated with J774 murine macrophages with and without DOX (i.e., CELVEC and DOX@CELVEC) was prepared by seeding cells into a 6-well plate at a density of 5 × 10^5^ cells per well. Cells were harvested following 4 h incubation and loaded with DOX as previously described in Section 2.2. Next, cells were labeled with FITC Annexin V as described by the manufacturer. Sample acquisition was performed using a BD LSRFortessa flow cytometer (BD Bioscience) and analyzed using FlowJo v 10 (BD Bioscience).

### Adhesion to inflamed endothelium in flow

Human umbilical vein endothelial cells (HUVEC, Lonza) were seeded into fibronectin-coated μ-Slide I^0.4^ Luer (Ibidi) at a concentration of 3 × 10^5^ cells /slide. Cells were maintained in endothelial basal media-2 supplemented with EGM-2 SingleQuots (Lonza) media and activated using TNFα (50 ng/mL) prior to treatment. Following overnight activation, an equal amount of CELVEC (generated with Jurkat T lymphocytes) were introduced into the flow chamber and allowed to incubate for 15 min. Following incubation, slides were gently washed to remove unbound cells and subjected to flow at a rate of 0.1 dyne/cm^2^ for 30 min. Microscope images were collected before and after flow to quantify percent bound using a Nikon Eclipse 80i equipped with an Andor monochrome camera and analyzed using NIS Elements software.

### Animal care

All animal studies were performed in accordance with the guidelines outlined in the Animal Welfare Act and the Guide for the Care and Use of Laboratory Animals. All protocols were approved by the Institutional Animal Care and Use Committee at the Houston Methodist Research Institute. Female athymic nude mice (4–6 week old) were purchased from Charles Rivers Laboratories and maintained as previously described^22^.

### Intravital microscopy

Mice were administered a retro-orbital injection of 1 × 10^6^ DOX@CELVEC and subsequently administered a retro-orbital injection of 40 kDa FITC-dextran tracer (1 mM, Invitrogen) for vasculature delineation prior to organ retrieval. Imaging was performed using a Nikon A1R laser scanning confocal microscope modified for intravital microscopy. Organs of interest were imaged *ex vivo* across multiple field-of-views and images were analyzed using NIS Elements software to determine maximum percent covered per organ. Sum of average area fraction was determined and divided across all organs for that time point.

### *In vivo* efficacy

Bioware® Brite Cell Line LL/2 Red-FLuc (Perkin Elmer, Inc) (1 × 10^6^ cells in 100 μL PBS) were implanted through tail vein injection into nude mice (n = 10). Five days after injection, mice were randomized by tumor size determined by bioluminescence using the Xenogen IVIS Imaging System (Caliper Life Sciences). Mice were injected through the lateral tail vein with free DOX (10 mg/kg), DOX@CELVEC (10 mg/kg DOX equivalent), or PBS (CTRL) every other day. At the indicated times, the animals were sacrificed by exsanguination and blood was collected. Upon completion, lung, heart, liver, and spleen samples were collected, weighed, and fixed in 10% buffered formalin overnight at 4 °C, transferred to 70% ethanol at 4 °C, and paraffin embedded for histological analysis.

### Statistical analysis

Statistics were calculated using GraphPad Prism software. Statistics for DOX loading and release were obtained with a nonlinear regression analysis using a one-phase association equation and least squares fit. Statistics for *in vitro* toxicity and *in vitro* dynamic flow assay were obtained using a one-way ANOVA followed by a Dunnett post-test. Statistics for *in vivo* tumor inhibition were obtained using a two-way ANOVA followed by a Dunnett post-test. Graphs are presented as a mean ± SEM. Probabilities are denoted as ****P ≤ 0.0001, ***P ≤ 0.001, **P ≤ 0.01 and *P ≤ 0.05.

## Results

### Generation of CELVEC

**Figure 2:**
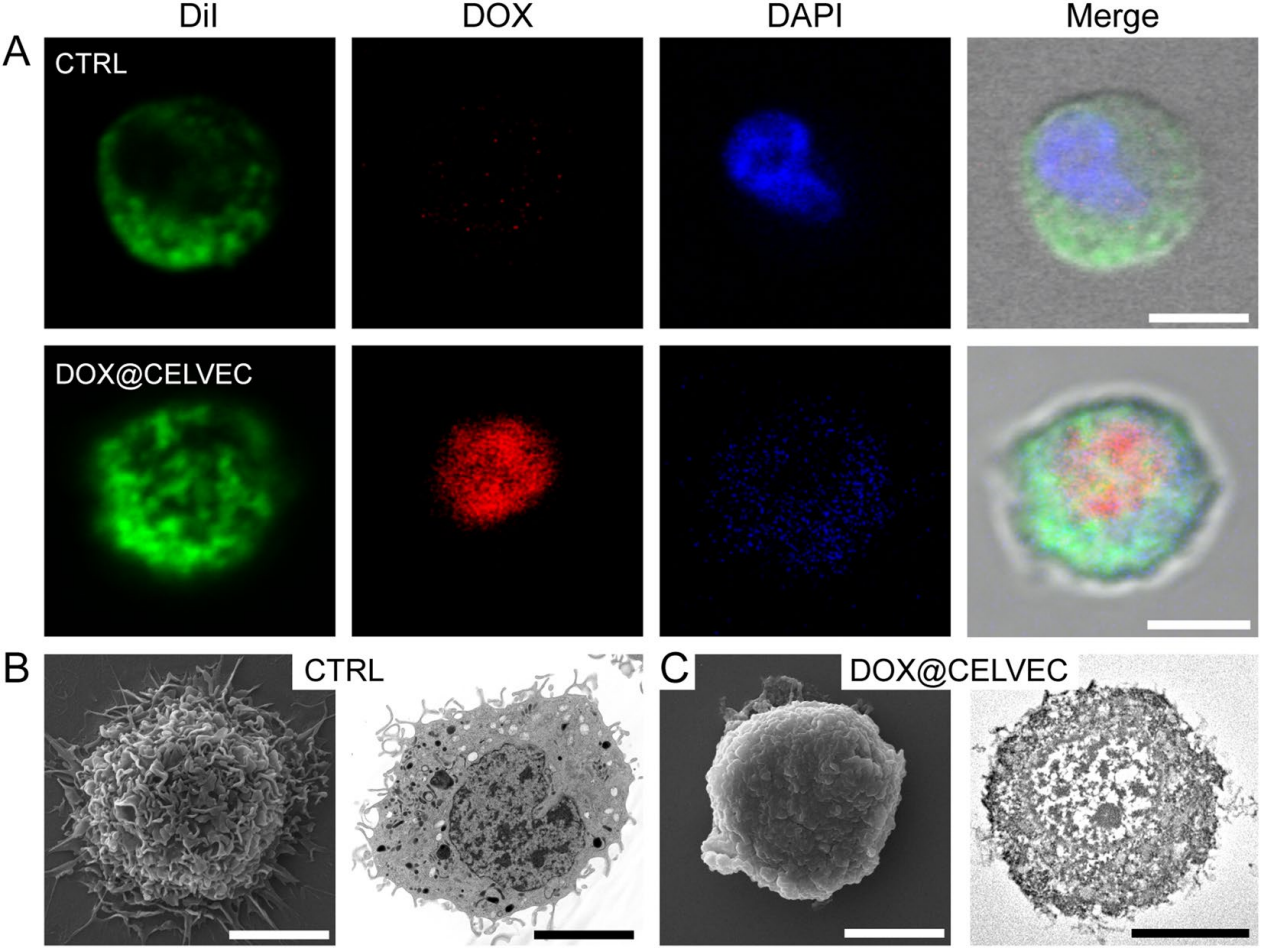
Fabrication of CELVEC. (**A**) Confocal microscope images of untreated murine macrophages (CTRL) and DOX@CELVEC depicting DiI surface membrane staining (green), DOX loading (red), and DAPI nucleus staining (blue). (**B**) Scanning and transmission electron microscope images of CTRL macrophages and (**C**) CELVEC. Scanning electron images shown on left, transmission electron images shown on right. Scale bars, 5 μm.

J774 murine macrophage cells were chosen as the cellular model for the generation of CELVEC for two primary reason: (i) extensive experience among our group using leukocyte-based models and (ii) to confirm previous work in this innovative field of drug delivery^18^. Contrasted to the pioneering work of Zhang and coworkers^18^, CELVEC were generated via electroporation to favor loading with free drug^23^ and do not rely on cellular activity to delivery their payload (Fig. 1). To enhance loading efficiency, DOX was first dissolved in an acidified electroporation buffer (pH = 5.2) to increase its solubility (Supplementary Fig. S1). At pH greater than 5.2, saturated DOX (20 mg/mL) resulted in nano- and micro-sized aggregates as indicated by an increase in polydispersity index (PDI, >0.637). As such, to maintain maximum solubility of DOX^24^, all further experiments were performed at pH 5.2. Following loading, confocal analysis exhibited the presence of DOX within the cell body with localization observed within the cell nucleus (i.e., DOX@CELVEC, Fig. 2A and Supplementary Fig. S2). Compared to non-electroporated control macrophages (CTRL), drug loading of CELVEC resulted in a reduction of cell size and a restructuring of the cell surface as demonstrated by scanning electron microscopy. Further assessment using transmission electron microscopy revealed a restructuring of the cell morphology and the presence of DOX within the nuclear environment (Fig. 2B,C and Supplementary Fig. S3).

### CELVEC loading and release

**Figure 3:**
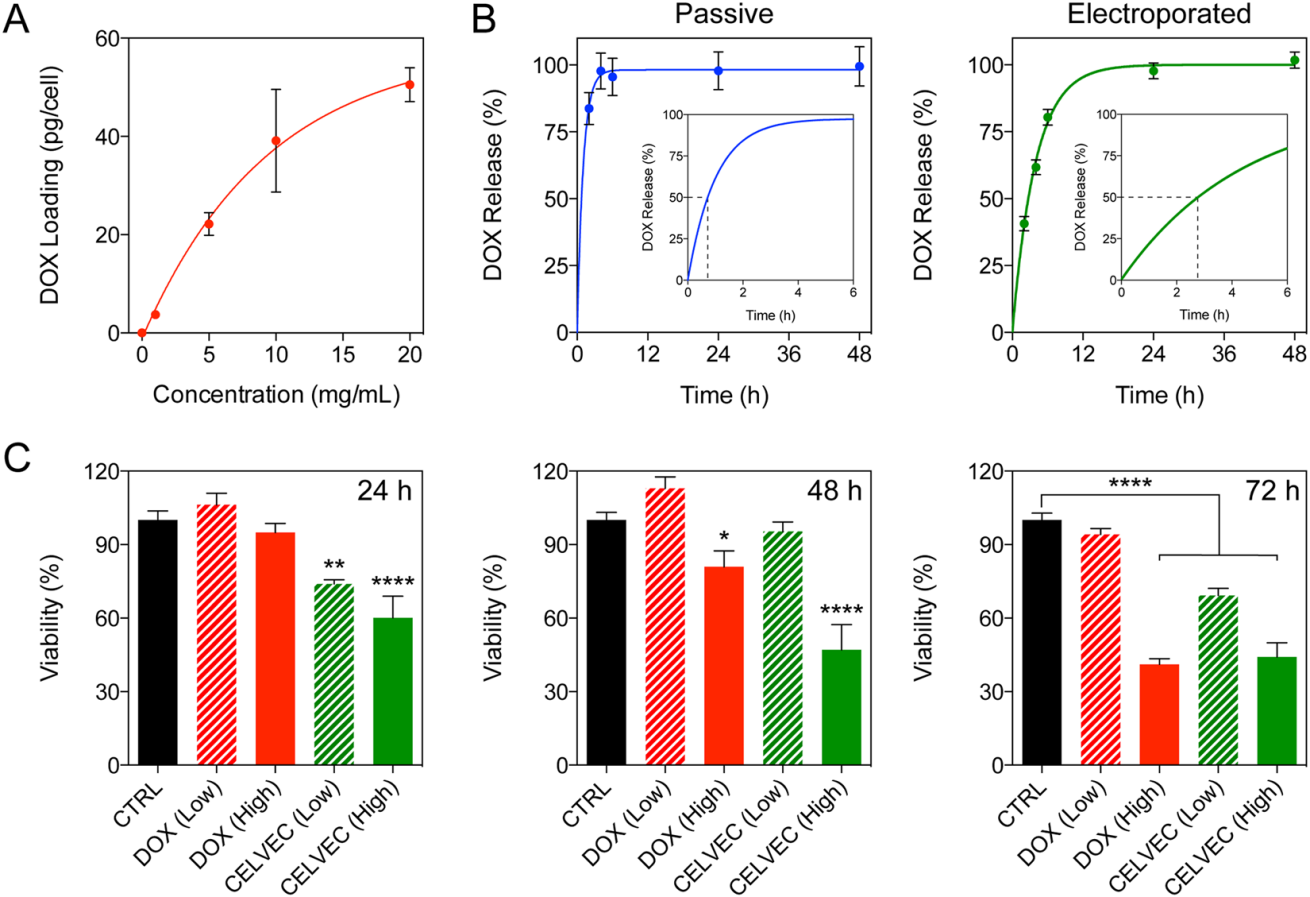
CELVEC as a drug delivery vehicle. (**A**) Loading of doxorubicin (DOX) into CELVEC as a function of initial DOX concentration used. (**B**) Release of DOX from macrophages over 24 h when loading was performed passively (left) or supplemented with electroporation (right). Inset graphs depict DOX release over 6 h as determined using a one-phase association ordinary fit line. Dotted black line designated 50% release of payload. (**C**) Cellular viability of human MDA-231 triple negative breast cancer cells following treatment with free DOX and DOX-loaded CELVEC at low (325 nM) and high (975 nM) dosages. The data is plotted as the mean ± SEM. *p < 0.05; **p < 0.01; ****p < 0.0001.

Electroporation of cells resulted in loading efficacy directly proportional to the concentration of DOX in solution, peaking at 50 pg/cell when using a 20 mg/mL DOX loading concentration (Fig. 3A). Calculation of loading efficiency as a function of total DOX loaded into the cells, therefore, was found to peak at 5–10 mg of DOX/mL. Compared to passive loading, electroporation increased the loading yield of DOX as demonstrated by flow cytometry (Supplementary Fig. S4). Release of DOX from DOX@CELVEC was next investigated over 48 h and compared to cells passively-loaded via a 10 min DOX incubation (20 mg/mL). While both systems exhibited a burst release occurring within the first 6 h, electroporated cells (i.e., DOX@CELVEC) displayed a delay in payload release when compared to passively-loaded cells. Specifically, passively loaded cells released 80% of their payload by 2 h while electroporated cells reached similar values only after 6 h (Fig. 3B). For this reason, electroporation was considered a critical step to achieve sustained drug delivery in the lungs following intravenous administration of DOX@CELVEC.

Next, to assess DOX@CELVEC’s potential in efficiently inhibiting cancer cell growth, we compared CELVEC cytotoxicity on human triple negative breast cancer cells (i.e., MDA-MB-231) to free DOX using concentrations previously reported in literature^25–28^. The cells were treated with both low (325 nM) and high (975 nM) concentrations of free DOX and DOX@CELVEC for 24, 48 and 72 h. Following a single-dose treatment as previously reported^29^, both low and high DOX concentrations exhibited an increase in cell toxicity when incorporated into CELVEC relative to free DOX (Fig. 3C). The cytostatic effect of free DOX and DOX@CELVEC were similar at 72 h, indicating free DOX possesses a similar internalization index as DOX released from a DOX@CELVEC treatment when given enough time. This is likely attributed to the increased efficiency of DOX as a result of DOX@CELVEC sedimentation, thereby resulting in an increase in DOX concentration in the vicinity of adhered cells.

To effectively characterize this phenomenon, CELVEC and DOX@CELVEC were administered to human breast cancer cells with or without a cell culture insert to inhibit direct contact between cells and CELVEC. In this experiment, the control was represented by CELVEC (i.e., electroporation with no DOX) administered similar to DOX@CELVEC (Supplementary Fig. S5). As expected, following 24 h of DOX@CELVEC administration, an evident cytostatic effect was demonstrated without a cell culture insert. This confirms that the previous increase in cytotoxicity was a result of the direct interactions of DOX@CELVEC and breast cancer cells.

### Effect of the loading of the chemotherapeutic on CELVEC viability

**Figure 4:**
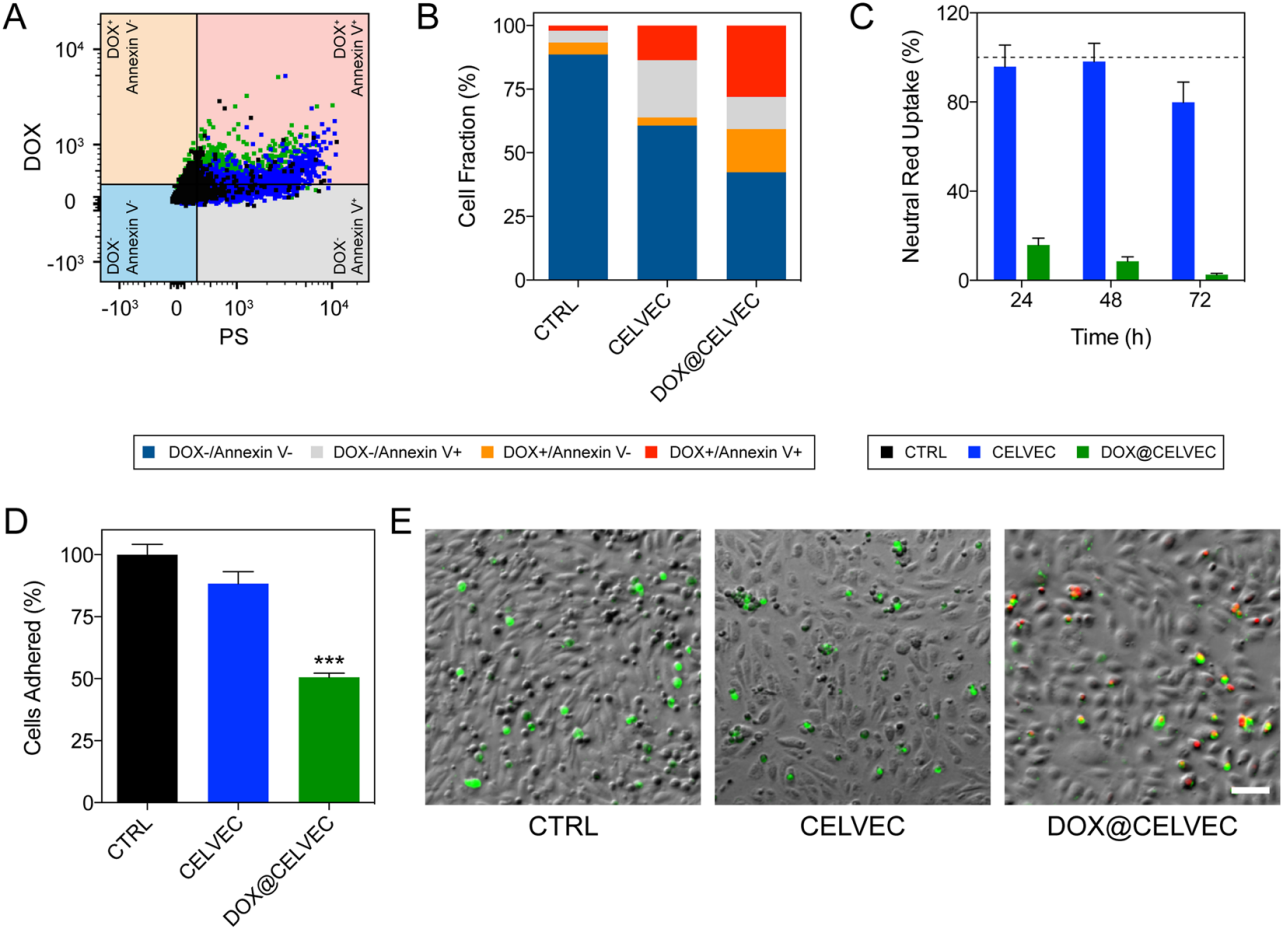
Effect of DOX loading in CELVEC performance. (**A**) Flow cytometry dot plot depicting PS on CELVEC (blue) and DOX@CELVEC (green) surface 4 h post-generation. (**B**) Quantitative assessment of flow cytometry dot plot data exhibiting DOX and Annexin V positive cells using gates established in (A). (**C**) Cytotoxic evaluation of murine macrophage cells following electroporation (CELVEC, blue) and electroporation supplemented with DOX (DOX@CELVEC, green) to create DOX@CELVEC. Neutral red uptake was employed to evaluate cellular viability and proliferation over 72 h. Doxorubicin loading was performed at 20 mg/mL. Values normalized to untreated murine macrophages (CTRL). (**D**) Quantification of CELVEC adhered to TNF-α-activated HUVEC following 30 min of *in vivo*-like flow conditions of 0.1 dyn/cm^2^. (**E**) Representative fluorescent microscope images used for (**D,E**) depicting CELVEC adhesion following 30 min. of flow. Scale bar, 100 μm. The data is plotted as the mean ± SEM. ***p < 0.001.

As previously mentioned, cell viability was not considered an important parameter in the design of CELVEC. However, it is worth noting poor viability could favor immune clearance as a result of an increase in the “eat-me” signal, phosphatidylserine (PS). To verify this hypothesis, we assessed the expression of PS on the surface 4 h following CELVEC generation (Fig. 4A and Supplementary Fig. S6). Quantification of the respective quadrant from Fig. 4A revealed CELVEC and DOX@CELVEC resulted in a ~6-fold increase in PS-expression cells relative to non-electroporated CTRL cells (Fig. 4B). Cytotoxicity test performed at 24, 48, and 72 h following generation of DOX@CELVEC resulted in a significant impact on cell viability while electroporated cells maintained consistent metabolic activity (Fig. 4C). However, despite the subjugation of our system to electroporation, adhesion surface proteins remained intact. Specifically, we evaluated the presence of critical surface protein markers (i.e., CD11a and CD11b) involved in the multi-step leukocyte diapedesis process (Supplementary Fig. S7). These proteins have previously been showcased by our group to maintain bioactivity following the isolation from cell membrane and incorporation into biomimetic carriers^3,4,16,17,30^. To this end, we next evaluated the retention and ability of CELVEC to adequately adhere to an activated endothelium monolayer under dynamic flow conditions (Fig. 4E,F). This data exhibited that adhesion properties, relative to untreated cells, were not completely lost following drug loading.

### CELVEC efficacy in favoring pulmonary delivery of the chemotherapeutic

**Figure 5:**
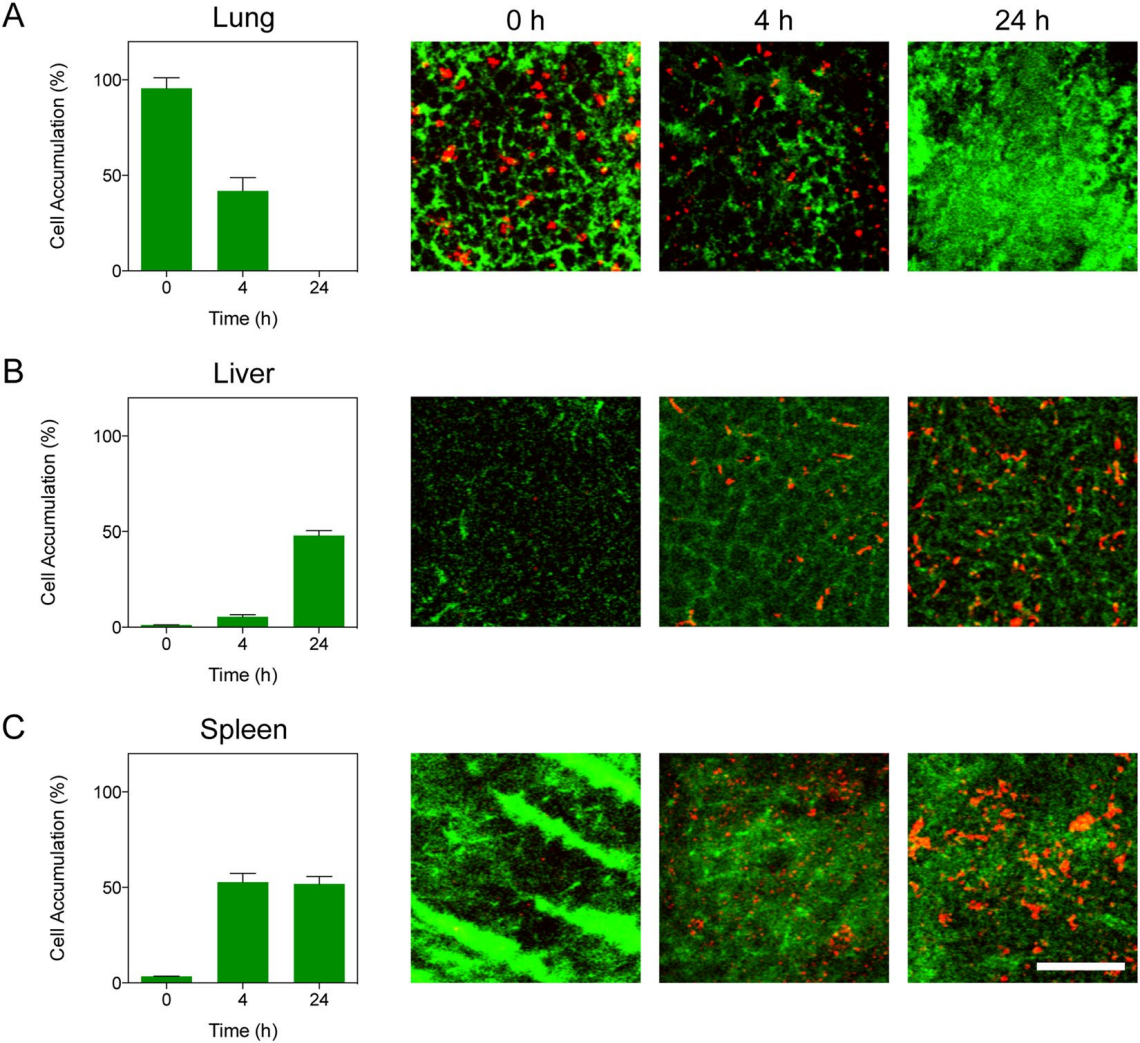
Biodistribution of DOX@CELVEC using intravital microscopy. Quantitative *ex vivo* analysis at 0, 4, and 24 h of accumulated DOX@CELVEC in (**A**) lung, (**B**) liver, and (**C**) spleen following intravenous administration. Representative intravital microscope images are displayed. Scale bar, 100 μm. Total accumulated DOX@CELVEC is presented as a percentage of all three organs at each time point. The data is plotted as mean ± SEM.

To evaluate pulmonary resident time of DOX@CELVEC, we first assessed biodistribution using intravital microscopy following administration to non-tumor-bearing mice (Fig. 5). Immediately following intravenous administration, *ex vivo* organ analysis revealed lungs initially retained ~96% of injected cells with a 2.2-fold reduction occurring at 4 h (Fig. 5A). Evaluation at 24 h post-administration resulted in less than 1% of DOX@CELVEC present in the lungs, indicating the highest accumulation occurs within the first 4 h and demonstrating clearance of our system by liver and spleen (Fig. 5B,C). This data depicted similar trends to CELVEC without DOX (Supplemental Fig. S8). Specifically, initial accumulation in the lungs with increasing sequestration by liver and spleen at later time points.

**Figure 6:**
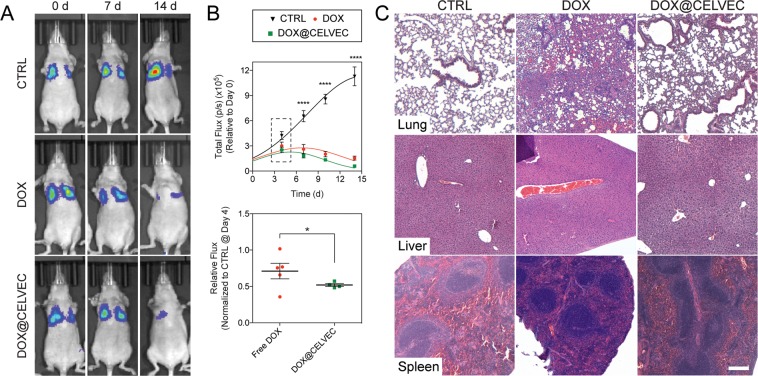
Efficacy and biocompatibility of DOX@CELVEC. (**A**) Whole body longitudinal non-invasive bioluminescent imaging of mice at 0, 7, and 14 days following inoculation with an LL/2 lung tumor model and administration of PBS (CTRL), DOX, or DOX@CELVEC every other day. (**B**) Quantitative assessment of total bioluminescent signal observed from tumor growth at 4, 7, 10, and 14 days post-treatment (top). Further comparison of relative flux normalized to CTRL was performed at 4 d (bottom). (**C**) Histological evaluation of lung, liver, and spleen following 24 h treatment with PBS (CTRL, left column), DOX (middle column) and DOX@CELVEC (right column). Scale bar, 200 μm. The data is plotted as mean ± SEM. *<0.05; **p < 0.01; ****p < 0.0001.

Next, assessment of DOX@CELVEC ability to inhibit tumor growth was evaluated using a lung cancer tumor model. Mice administered either free DOX or DOX@CELVEC every other day demonstrated a 20-fold reduction in tumor signal following two weeks of treatment with a significant difference observed between free DOX and DOX@CELVEC in as little as 4 d (Fig. 6A,B). Histopathological evaluation of liver and spleen revealed minimal differences for both treatment groups when compared to intravenously administered CTRL macrophages (Fig. 6C). However, lungs displayed severe inflammation and collapsed alveoli due to free DOX treatment, while DOX@CELVEC displayed no observable differences relative to CTRL. Next, we evaluated cardiac tissue to assess the ability of CELVEC-mediated delivery to mitigate cardiotoxicity. Our analysis revealed no substantial cardiac tissue damage was observed for DOX@CELVEC while free DOX exhibited minor inflammation (Supplementary Fig. S9).

## Discussion

Current nanotherapeutics are designed to exploit the unique characteristics of cancer diseases such as the enhanced permeability and retention effect and/or the presence of surface receptors that are overexpressed in tumor cells^31,32^ or rationale fabrication design^33^. Targeted drug delivery platforms are often developed by employing complex synthetic procedures resulting in low yield, costly fabrication^34^, and potential immunoreactivity^17^. Specifically, the high cost associated with the fabrication of synthetic systems represents a significant obstacle in the widespread use of nano-based therapies in the clinical setting^35,36^. To this end, we believe research efforts aimed at mitigating production costs while also generating innovative nanomaterials and surface materials are critical. However, targeted-based delivery is not often the primary choice of treatment due to the difficulty associated with reaching disseminated cancer^37^.

A promising strategy to overcome these limitations could be achieved by shifting the targeting mechanism from the cell to the organ level. As such, the organs of the MPS represent an ideal model to evaluate this hypothesis, with the lungs representing a well-known site of primary and metastatic tumors. Inspired by the latest trends in biomimetic medicine^15,18^, we generated a drug delivery system using macrophages as delivery vehicles, harnessing our groups extensive knowledge on this platform^3,4,16,17,30^. Specifically, CELVEC were strategically engineered to incorporate a therapeutic payload using a simple, electroporation-guided loading procedure.

The specific novelty of our system is owed to the implementation of fabrication parameters that provide us with a delivery system capable of achieving controlled release of a payload (i.e., <6 h). Previous work^38^ demonstrated that therapeutics, biologics, and nanoparticles can be easily loaded within the cell cytoplasm via electroporation, inspiring us to generate pH-tuned DOX to maximize DOX loading within the cell (i.e., 50–60 pg DOX/cell). More importantly, the release time window corresponded to the residence time of CELVEC in the pulmonary environment (i.e., 4–6 h) with significant accumulation in liver and spleen occurring beyond 8 h. In other words, CELVEC resulted in a controlled release up to 6 h and were effective in increasing drug concentration in the pulmonary tissue.

This is likely attributed to the electroporation of DOX crystals enabling prolonged drug release, although we speculate that this phenomenon can also be driven by the chemotherapeutics’ high affinity for cellular compartments. These compartments could potentially serve as storage compartments for the payload, driven by the intercalation with nucleic acids without covalent bonding. A similar approach was shown successful in the loading of enucleated cells (i.e., erythrocytes^39^), where fixation agents were used to prolong drug release^40^.

In the process of optimizing drug encapsulation efficiency, maintaining long-term cell viability was not a critical component. Specifically, as our primary objective was to harness the advantages provided by the size of the system, biological function was not considered in the rationale design of our system. Despite this, we demonstrated electroporated cells maintained the expression of adhesion-based transmembrane proteins. In addition, as similarly depicted in our previous work^3,15–17,41^, the surface of CELVEC partially maintained its affinity for inflamed vascular as depicted by *in vitro* dynamic flow conditions, representing an advantage in the ability to target inflamed organs.

CELVEC further demonstrated favorable suppression of tumor growth in the lungs and did not display any significant acute effects in major MPS organs, thereby demonstrating the biocompatibility of our system. However, despite a clear advantage in enhancing the cytostatic properties of the therapeutic, more work is necessary to evaluate cardiotoxicity. While slight inflammation was observed in free DOX when compared to DOX@CELVEC, higher doses are needed to properly assess cardiotoxic potential. All considered, when compared to traditional attempts to exploit size as a targeting strategy for drug delivery, our method represents a low-cost, efficient solution to generate effective pulmonary drug delivery vehicles.

## Conclusions

In conclusion, we demonstrated the ability to favorably employ electroporation in the development of cell-based delivery systems. In addition, despite foregoing preservation of biological functions as showcased by a decrease in cell viability, CELVEC were capable of providing sustained payload release for up to 6 h, paralleling residence time in lungs prior to clearance. Future efforts will be focused on: (i) assessing our electroporation-based loading strategy on other cell lines to achieve increased drug encapsulation, favorable release kinetics, and increased residence time in the lung microenvironment (ii) examining other therapeutics as a function of their hydrophobicity and electroporated-mediated loading potential, (iii) efficacy in treating pulmonary-based diseases, (iv) determine surface properties of CELVEC that can favor cancer lesion vasculature targeting. To conclude, the development of delivery systems designed to target at the organ level have the potential to provide a unique avenue for generalized treatment. This is evident by the necessity for a payload to homogenously perfuse the organ as in the case of adjuvant-based treatment in cancer and infectious disease. As such, we demonstrate the use of micron-sized cellular systems have the potential to provide a viable treatment strategy for pulmonary-based diseases.

## Supplementary information

Supplementary Information
