# Supplementary material for: Biomimetic cellular vectors for enhancing drug delivery to the lungs
Source: Sci Rep. 2020 Jan 13;10:172. doi: 10.1038/s41598-019-55909-x (PMC6957529; doi:10.1038/s41598-019-55909-x)
Supplement: Supplementary file 1 — Supplementary Information [file 41598_2019_55909_MOESM1_ESM.pdf]

SUPPLEMENTARY INFORMATION

Biomimetic cellular vectors for enhancing drug delivery to the lungs

Michael Evangelopoulos[1,#], Iman K. Yazdi[1,#], Stefania Acciardo[1,2], Roberto Palomba[1,3], Federica Giordano[1,4], Anna Pasto[1,5], Manuela Sushnitha[1,6], Jonathan O. Martinez[1], Nupur Basu[1], Armando Torres[1], Sarah Hmaidan[1], Alessandro Parodi[1,7,*], Ennio Tasciotti[1,8,*]

[1]Center for Biomimetic Medicine, Houston Methodist Research Institute, Houston, TX, USA.

[2]Department of Mechanical and Aerospace Engineering, Politecnico di Torino, Turin, Italy.

[3]CEINGE Biotecnologie avanzate, Naples, Italy

[4]School of Medicine and Surgery, University of Milano-Bicocca, Monza, Italy

[5]Veneto Institute of Oncology–IRCCS, Padua, Italy

[6]Department of Bioengineering, Rice University, Houston, TX, USA

[7] Institute of Molecular Medicine, Sechenov First Moscow State Medical University, Moscow, Russia

[8]Houston Methodist Orthopedic and Sports Medicine, Houston Methodist Hospital, Houston, TX, USA.

*To whom correspondence should be addressed:

Dr. Alessandro Parodi

Department of Pharmacology

College of Medicine

University of Illinois at Chicago

835 S Wolcott Ave E403

Chicago, IL 60612

aparodi@uic.edu

Dr. Ennio Tasciotti

Center for Biomimetic Medicine

Houston Methodist Research Institute

6670 Bertner Ave

Houston, TX, 77030

etasciotti@houstonmethodist.org

[#] These authors contributed equally to this work.

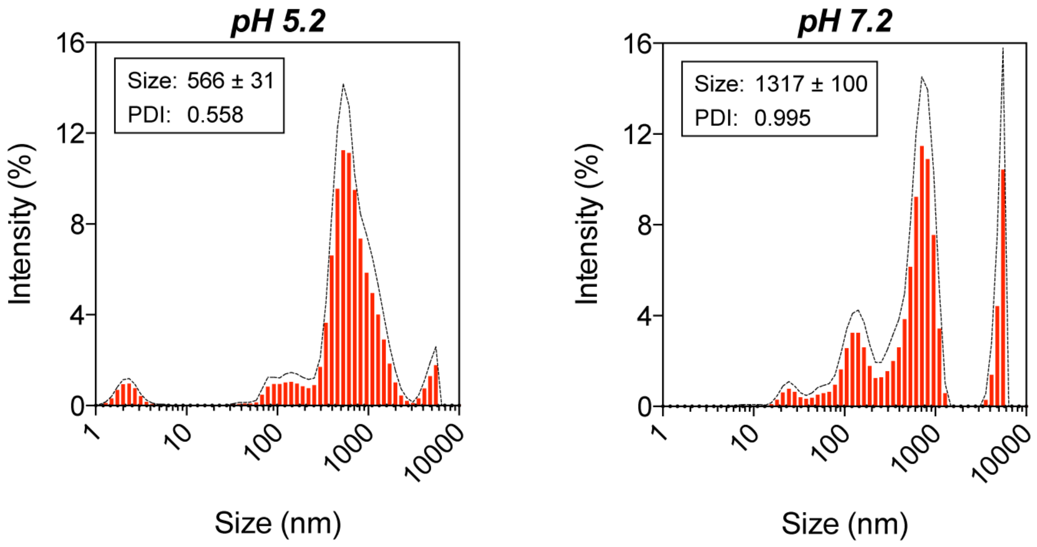

Figure S1. Dynamic light scattering analysis of doxorubicin suspended in electroporation buffer with pH 5.2 (left) and pH 7.2 (right). Dotted black line represents standard deviation (n=6). Inset depicts size as a unit of nm with PDI representing polydispersity index.

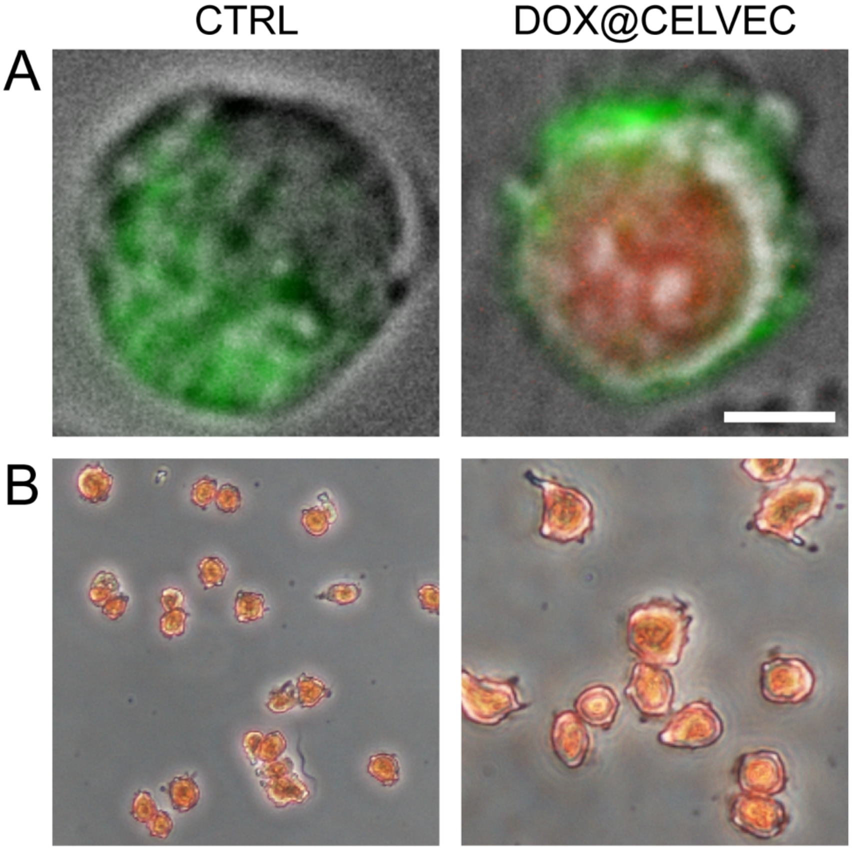

Figure S2. (A) Fluorescent microscope image depicting stained cellular membrane (green) and DOX (red) distribution of DOX@CELVEC. Scale bar, 10 μm. (B) Low (left) and high (right) magnification optical microscope images following generation of DOX@CELVEC.

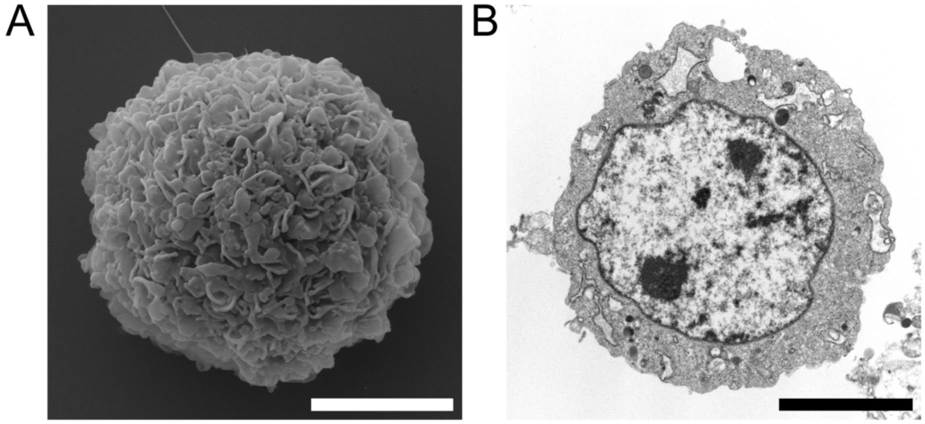

Figure S3. (A) Scanning electron and (B) transmission electron micrographs demonstrating J774 murine macrophage following electroporation to create CELVEC. Scale bars, 5 μm.

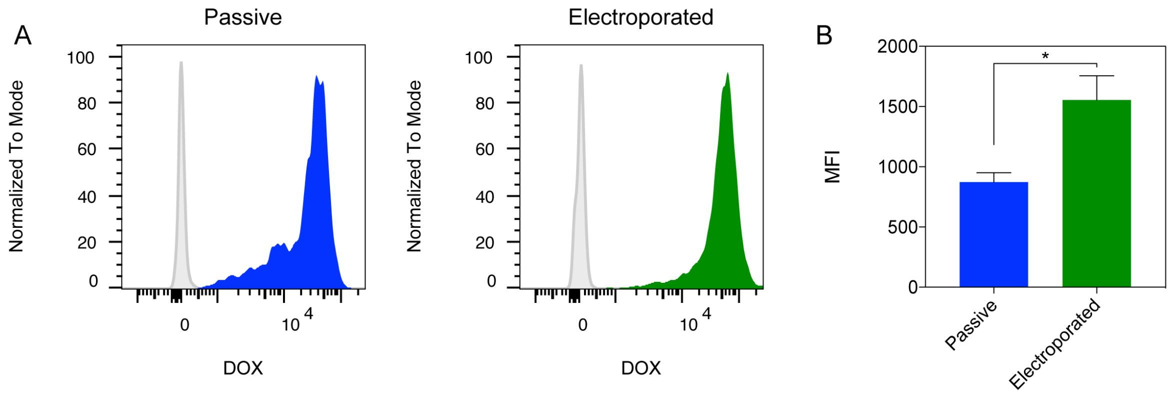

Figure S4. (A) Flow cytometric histogram profile depicting control cells and either passive (left) or electroporated-mediated (right) loading of DOX. (B) Quantitative analysis of mean fluorescence intensity (MFI) or passive and electroporated following normalization to the respective empty cells. The data is plotted as the mean ± SEM. * $p < 0.05$.

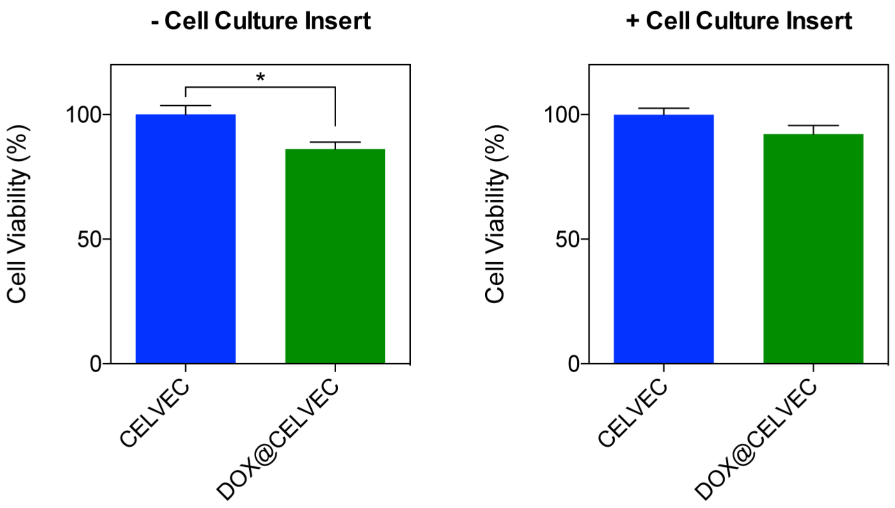

Figure S5. Cytotoxic evaluation of on MDA-MB-231 breast cancer cells following 24 h treatment with CELVEC or DOX@CELVEC without (left) or with (right) the use of cell culture inserts. The data is plotted as the mean ± SEM. * $p < 0.05$.

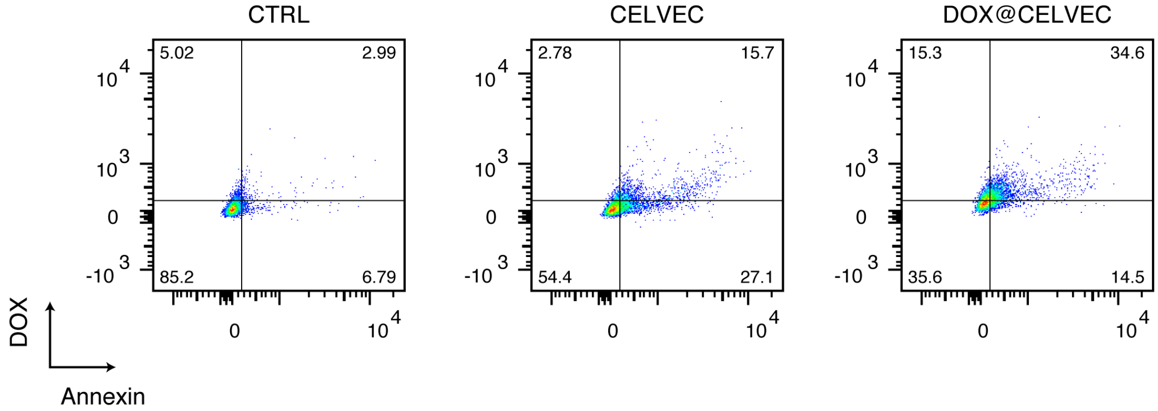

Figure S6. Individual dot plot graphs of data plotted in Figure 4A. Flow cytometry gates set in accordance with CTRL cells. Assessment was performed using FlowJo v X.

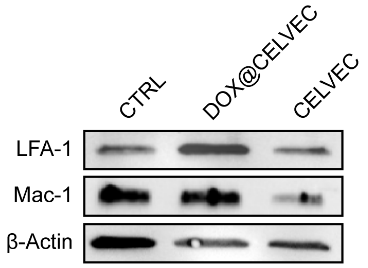

Figure S7. Western blot analysis of CELVEC and DOX@CELVEC to demonstrate key surface protein expression of LFA-1 and Mac-1 following electroporation.

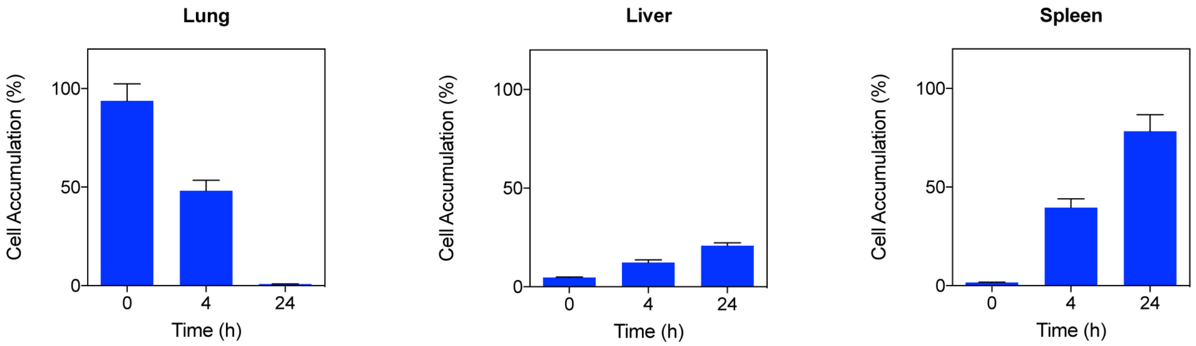

Figure S8. *Ex vivo* intravital microscopy analysis of lung, liver, and spleen following intravenous administration of CELVEC at 0, 4, and 24 h. The data is plotted as the mean ± SEM.

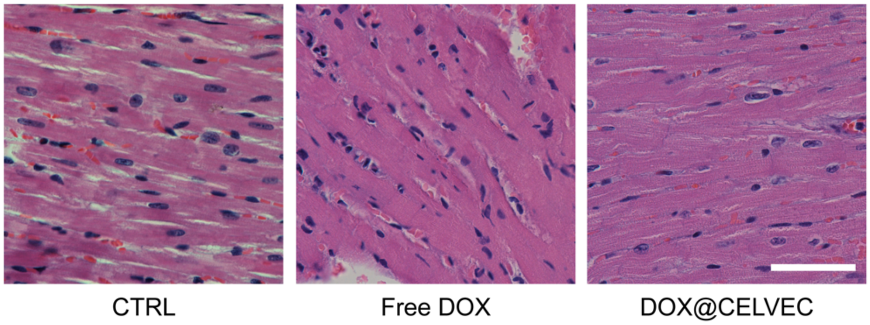

Figure S9. Representative histopathological tissue sections of mouse hearts following 24 h administration of Free DOX or DOX@CELVEC compared to an untreated control (CTRL) section. Scale bar, 50 um.
